# Editorial: Ultra-processed foods: impacts on diet quality, health, consumer behavior, and food systems

**DOI:** 10.3389/fpubh.2026.1837261

**Published:** 2026-05-15

**Authors:** Maria Alvim Leite, Yeji Baek, Flávia dos Santos Barbosa Brito, Carla Gonçalves, Débora Martins dos Santos, Amanda Rodrigues Amorim Adegboye

**Affiliations:** 1 Department of Preventive Medicine, University of São Paulo Medical School, São Paulo, SP, Brazil; 2 Department of Primary Health Care, Faculty of Medical Sciences, Santa Casa de São Paulo, São Paulo, SP, Brazil; 3 School of Public Health and Preventive Medicine, Faculty of Medicine, Nursing and Health Sciences, Monash University, Melbourne, VIC, Australia; 4 Health Economics and Policy Evaluation Research, Faculty of Pharmacy and Pharmaceutical Sciences, Monash University, Parkville, VIC, Australia; 5 Department of Social Nutrition, Nutrition Institute, Rio de Janeiro State University, Rio de Janeiro, RJ, Brazil; 6 School of Life and Environmental Sciences (RISE-HEALTH), University of Trás-os-Montes e Alto Douro, Vila Real, Portugal; 7 Centre for the Research and Technology of Agro-Environmental and Biological Sciences, University of Trás-os-Montes e Alto Douro, Vila Real, Portugal; 8 Royal College of Nursing, Institute of Nursing Excellence, London, United Kingdom

**Keywords:** diet quality, non-communicable disease, nutrition, public health, ultra-processed food

Over recent decades, the consumption of ultra-processed foods (UPFs) has increased substantially worldwide, becoming a dominant component of dietary patterns in many countries (1). This trend has paralleled the rising prevalence of obesity and other non-communicable diseases, including cardiovascular diseases, type 2 diabetes, and mental health disorders (2). Although these trends are driven by complex and multifactorial processes, the growing centrality of UPFs in contemporary food systems has emerged as a key concern for public health.

In parallel with these epidemiological shifts, the scientific production on UPFs has expanded rapidly. A robust body of evidence, including large cohort studies and systematic reviews with meta-analysis, has consistently documented associations between higher UPF consumption and a range of adverse health outcomes, such as weight gain, cardiometabolic risk, all-cause mortality, and mental health conditions (3, 4).

In response to this accumulating evidence, international organizations have incorporated or are actively discussing recommendations to reduce UPF consumption within policy frameworks (5, 6). Also, food-based dietary guidelines from countries such as Brazil, Uruguay, Peru, Ecuador, Mexico, Chile, Colombia, Israel, Malaysia, India, France, and Canada have explicitly emphasized limiting UPFs and prioritizing fresh or minimally processed foods (7). Beyond dietary recommendations, a growing set of public policy strategies has been proposed and, in some contexts, implemented. These include front-of-package warning labels, taxation of unhealthy products, restrictions on marketing (especially to children), reformulation policies, and interventions to improve food environments. Together, these efforts reflect a shift toward addressing the structural and commercial determinants of unhealthy diets (8).

Within this context, the Research Topic “*Ultra-Processed Foods: Impacts on Diet Quality, Health, Consumer Behavior, and Food Systems*” was conceived to advance understanding of the multifaceted relationships between UPF consumption and health. This Research Topic aimed to elucidate how UPFs interact with dietary quality, consumer behavior, and broader food systems, welcoming interdisciplinary contributions spanning nutrition, public health, psychology, sociology, economics, and policy research.

The 13 articles included in this Research Topic offer a diverse and complementary body of evidence, spanning multiple regions of the globe. A substantial contribution comes from the Americas—including studies conducted in Brazil, Colombia and the United States, as well as cross-national analyses across the region (José et al.; Grilo et al.; Castrillón-Ruiz et al.; Lee et al.; Rose and Kolodinsky; Rossi et al.; Evans et al.; Barb et al.; Vatavuk-Serrati et al.). Additional evidence is drawn from Asia, with data from China (Li et al.), and from Europe/West Asia, including a study conducted in Istanbul, Türkiye (Akin et al.). The issue also includes a narrative review synthesizing findings from diverse global contexts (Mottis et al.) and a conceptual commentary addressing broader debates in nutrition science (Ullian).

Several studies examined associations between UPF consumption and health outcomes across different populations and life stages. Findings include links between UPF consumption and poorer diet quality and elevated glucose levels in adolescents (Castrillón-Ruiz et al.), potential impairments in executive cognitive function among older adults (Lee et al.), and associations with female infertility mediated by body mass index (Evans et al.). A narrative review further highlights the potential long-term consequences of UPF exposure during critical periods such as pregnancy, childhood, and adolescence, particularly in relation to brain development and mental health (Mottis et al.). In addition, research among shift workers underscores the role of psychological factors—such as stress, anxiety, and hedonic hunger—in shaping UPF consumption patterns (Akin et al.).

Other contributions explored the social and behavioral determinants of UPF consumption. Studies examining university students and immigrant populations revealed how social vulnerability and acculturation processes influence dietary patterns, often increasing reliance on UPFs while reducing consumption of fresh foods (José et al.; Barb et al.). Research on children and adolescents emphasized the importance of peer and family environments in shaping the consumption of ultra-processed beverages (Li et al.). Complementing this, evidence on consumer perceptions suggests that awareness of UPFs is associated with a shift in how individuals define healthy foods, with greater emphasis on processing-related attributes beyond traditional nutrient-based criteria (Rose and Kolodinsky).

At the food system level, one study documented the high prevalence of UPFs in newly launched products across the Americas, highlighting the structural drivers of exposure to these foods (Vatavuk-Serrati et al.). Another contribution evaluated the effectiveness of a behavioral economics intervention in food pantries, showing limited impact on reducing the selection of UPFs—underscoring the challenges of modifying food choices in constrained environments (Rossi et al.). Methodological advances are also represented, with a study proposing replicable ingredient-based approaches to classify UPFs in large food databases, supporting both research and regulatory applications (Grilo et al.). Finally, a conceptual paper addresses ongoing debates within nutrition science, examining the tensions, power dynamics, and epistemological disputes surrounding the role of UPFs in dietary recommendations (Ullian).

Taken together, the studies in this Research Topic reinforce the understanding of UPFs as a complex and multidimensional public health issue. They highlight the interplay between biological, psychological, social, and structural factors, as well as the need for integrated and interdisciplinary approaches. Importantly, they also point to persistent gaps in knowledge—particularly regarding mechanisms, effective interventions, and policy implementation—that warrant further investigation.

As UPFs continue to reshape global diets, advancing evidence, strengthening policies, and fostering interdisciplinary dialogue remain essential. We hope this Research Topic contributes to these efforts by providing new insights and stimulating future research and action aimed at improving population health and transforming food systems.
